# Hacia el envejecimiento saludable en América Latina y el Caribe: ¿no dejar a nadie atrás?^*^

**DOI:** 10.26633/RPSP.2021.120

**Published:** 2021-09-13

**Authors:** Norah C. Keating, Leocadio Rodríguez Mañas, Andrés De Francisco

**Affiliations:** 1 International Association of Gerontology and Geriatrics Nueva York Estados Unidos de América International Association of Gerontology and Geriatrics, Nueva York, Estados Unidos de América; 2 Hospital Universitario de Getafe Getafe España Hospital Universitario de Getafe, Getafe, España; 3 Organización Panamericana de la Salud, Washington, DC Estados Unidos de América Organización Panamericana de la Salud, Washington, DC, Estados Unidos de América

**Keywords:** Anciano, envejecimiento saludable, América Latina, Región del Caribe

El marco de envejecimiento saludable ha creado una estructura para la investigación a través de la Década del Envejecimiento Saludable de las Naciones Unidas (2021-2030), así como para la acción destinada a abordar la misión de los Objetivos de Desarrollo Sostenible de “no dejar a nadie atrás” (1). Se insta a los investigadores, los gobiernos y la sociedad civil a que, a lo largo de la década, elaboren estrategias que permitan detectar y abordar las desigualdades y fomentar el envejecimiento saludable. Este programa requiere el esfuerzo coordinado de los investigadores en gerontología y geriatría para afrontar la amplia variedad de problemas sociales y de salud que se producen en el envejecimiento, incluidos los que afectan la capacidad funcional, la participación social y las necesidades de las personas mayores en el contexto de los sistemas de atención social y de salud (2).

El envejecimiento saludable es dinámico. Refleja procesos que se producen a lo largo de la última parte del curso de la vida, y se ve influido por factores como las capacidades mentales y físicas, el entorno y las relaciones entre ellos. Si se da una “adaptación adecuada”, el resultado es el bienestar (3) y oportunidades de que las personas mayores en todos los países y regiones sean y hagan aquello que tengan motivo para valorar (4). El marco de envejecimiento saludable exige que realicemos un seguimiento de cómo evoluciona el estado de salud con el transcurso del tiempo y cómo pueden influir los sistemas de salud en las trayectorias de salud. Se hace necesario llenar las lagunas existentes en el conocimiento respecto del apoyo prestado por el entorno familiar y el grado en el que las comunidades disponen de recursos suficientes para facilitar la vida a las personas mayores. La influencia de temas relevantes como el cambio climático y el edadismo debe llevarse al campo del discurso sobre el envejecimiento saludable. Es necesario elaborar indicadores de bienestar que reflejen la interrelación entre persona y entorno, y utilizarlos para fundamentar las políticas y las intervenciones prácticas destinadas a reducir las desigualdades.

Los artículos incluidos en este número especial contribuyen a la agenda mundial de investigación de la próxima década y constituyen una aportación importante al conocimiento actual sobre el envejecimiento saludable en América Latina y el Caribe. Varios de los artículos tratan la cuestión del estado funcional y la elaboración de modelos sobre la dependencia funcional futura. Abordan los componentes del envejecimiento saludable: las capacidades intrínsecas relacionadas con la salud física y mental, y las interacciones con entornos clave como el hogar, la comunidad y las políticas más amplias. En conjunto, arrojan luz sobre el grado en el que las personas mayores de la Región pueden alcanzar sus metas.

Martínez et al. plantean el contexto general del envejecimiento al presentar las tendencias que se han producido en los últimos 30 años en la esperanza de vida y la esperanza de vida saludable (5). Su trabajo explora la paradoja de los aumentos sustanciales de la esperanza de vida pero con aumentos más pequeños de la esperanza de vida saludable. Sus datos indican que gran parte de la enfermedad y la discapacidad que se producen en la etapa avanzada de la vida pueden prevenirse o controlarse. González-González y colaboradores llegan a conclusiones similares, y predicen un aumento en la prevalencia de la dependencia en México (6). Gómez et al. (7) agregan evidencia obtenida en Colombia sobre los factores predictivos de la dependencia en una etapa avanzada de la vida, como son la capacidad intrínseca, el entorno social y el entorno físico (especialmente el hecho de vivir en comunidades rurales). Tres artículos proponen soluciones mediante políticas para abordar las limitaciones evitables causadas por una enfermedad en una fase avanzada de la vida. Morsch et al. (8) usan estudios de casos en varios países para mostrar la eficacia de las intervenciones dirigidas a apoyar la autogestión, y afirman que proporcionar herramientas para el manejo de la salud de las personas mayores constituye un imperativo tanto económico como ético. Pérez-Zepeda et al. (9) ilustran cómo las estrategias de salud pública durante la pandemia de COVID-19 en México y Colombia han tenido una repercusión diferente en la mortalidad de las personas mayores. Alonso Bouzón et al. (10) argumentan sobre la eficiencia de adaptar para América Latina programas de intervención ya acreditados destinados a reducir la fragilidad, basándose en el ejemplo del programa ADVANTAGE que se usa en países de toda Europa.

Las familias y las redes sociales de las personas mayores son contextos importantes para el envejecimiento saludable. Esteve (11) documenta la composición de los hogares en 23 países, e indica que la mayoría de las personas viven con los hijos y otros familiares. Sin embargo, la presencia de familiares no siempre proporciona un apoyo. En su investigación en México, Orozco Rocha et al. (12) observaron una reducción de la prevalencia y la magnitud de las transferencias económicas de los hijos a sus progenitores de mayor edad en México con el transcurso del tiempo, y llegaron a la conclusión de que los hijos no eran capaces de reducir la desigualdad existente en las categorías con ingresos más bajos. En su investigación sobre personas mayores en los altos Andes, Parodi et al. (13) observaron que una movilidad limitada estaba asociada a un apoyo social insuficiente.

Varios autores presentan políticas y programas que se consideran importantes para abordar las desigualdades. En su documento sobre la situación de las personas mayores en Colombia, Tamayo Giraldo et al. (14) sostienen que es necesaria una política pública dirigida a abordar las brechas existentes en la participación ciudadana y las oportunidades laborales formales a objeto de aumentar la calidad de vida de las personas mayores. Estos autores observan que el mejoramiento de la seguridad en los ingresos constituye uno de los principales modos de alcanzar este objetivo. El trabajo de Barrientos (15) proporciona también datos sobre las desigualdades en toda América Latina, y señala que aproximadamente 85% de las personas de más de 65 años no tienen ninguna seguridad en cuanto a sus ingresos, en contraposición con el 15% de las personas más ricas, en las que la seguridad de los ingresos muestra un aumento brusco en la etapa avanzada de la vida. Los sistemas de atención primaria de salud y de atención a largo plazo se consideran también componentes esenciales de las respuestas de políticas para abordar las necesidades de las personas mayores. González-González et al. (6) presentan un conjunto de indicadores de la capacidad de respuesta del sistema de salud que puede usarse para el seguimiento de su efectividad, y Villalobos Dintrans et al. (16) subrayan las necesidades crecientes de atención a largo plazo en la Región. Estos autores proporcionan directrices para determinar el tipo de sistema de cuidados a largo plazo que es más apropiado para diferentes países, y señalan que los países deben tomar medidas antes de que sea demasiado tarde.

Esta serie de artículos proporciona una base sólida sobre la cual se puede construir el conocimiento sobre el envejecimiento en América Latina y el Caribe en el próximo decenio. Hay mucho por hacer para resaltar los dividendos que aporta la longevidad y para conseguir que sus beneficios se obtengan de forma equitativa en los distintos países y dentro de cada uno de ellos. La evidencia sobre desigualdades que se presenta en los artículos de este número, junto con la investigación adicional a realizar a lo largo del decenio, pueden servir de fundamento para políticas y sistemas destinados a aumentar la seguridad en los ingresos, el acceso a los servicios de salud, el apoyo a las personas mayores y sus familias y la mejora de la infraestructura en las comunidades que han quedado atrás a causa de su ubicación geográfica remota o su inestabilidad política. El fortalecimiento de la investigación sobre el envejecimiento saludable en América Latina y el Caribe nos aproximará, ciertamente, al objetivo de no dejar a ninguna persona mayor atrás.

## Declaración.

Los autores son los únicos responsables de los puntos de vista expresados en el manuscrito, que pueden no reflejar necesariamente la opinión o la política de la *RPSP/RAJPH* o de la OPS.
